# Cavitation during tensile deformation of isothermally crystallized polypropylene and high-density polyethylene

**DOI:** 10.1007/s00396-012-2789-5

**Published:** 2012-09-05

**Authors:** Andrzej Pawlak

**Affiliations:** Centre of Molecular and Macromolecular Studies, Polish Academy of Sciences, Sienkiewicza 112, 90-363 Lodz, Poland

**Keywords:** Polypropylene, Polyethylene, Cavitation, Tensile properties, Plastic deformation, Isothermal crystallization

## Abstract

The cavitation phenomenon was studied in isothermally and non-isothermally crystallized polypropylene and high-density polyethylene. It was found that nano-voids were not present in the crystallized samples, but were formed during their tensile deformation. The process of cavitation was initiated before reaching the yield point. The ellipsoidal voids were initially elongated perpendicularly to the deformation direction, but if the polymer (i.e., high-density polyethylene) was able to deform beyond the yield, then the reorientation of voids into the deformation direction was observed at local strains of 100–200 %. This behavior was similar to that observed previously in the samples crystallized without an exact control of solidification conditions. The calculations of Guinier’s radius showed that voids in deformed polypropylene samples were characterized by the gyration radii of 28–50 nm. Smaller voids were observed in polyethylene. The scale of cavitation during deformation, studied on the example of polyethylene, depended on the preceding crystallization process and was most intensive for the specimens crystallized at the highest temperature of 125 °C.

## Introduction

The cavitation phenomenon was discovered in many semicrystalline polymers. The generation of voids (cavities) is usually observed in two different circumstances: during isothermal crystallization [1–9] and during tensile deformation [10–14]. To my knowledge, so far, both cases have been analyzed and described separately. The reason of cavitation during isothermal crystallization is the existence of pockets of melt surrounded by growing spherulites. The volume of molten material in these pockets (called weak spots) is not sufficient for complete crystallization because the density of the crystalline phase is higher than the density of the molten polymer [2]. The negative (i.e., 3D tensile) pressure arises inside the weak spot with time, and as a result, the growth of neighbor spherulites becomes slower and the polymer melt is stretched. Estimations show that the negative pressure in polypropylene (PP) weak spots may be high, even 19–40 MPa [1, 3].

When the negative pressure reaches the level of cohesive strength of melt, then a rapid break of melt occurs and 1-μm or more micrometer voids are formed. The presence of a cavitation nucleus (e.g., impurities) supports the initiation of the process at lower pressure. Often, the fracture of melt is observed at the same time in adjacent weak spots, which suggests that the propagation of an acoustic wave emitted in the cavitation act, resulting from a sudden pressure change, induces the cavitation in other weak spots. The acoustic emission from the crystallizing polymer was recorded by Galeski et al. [4]. Cavitation relaxes stresses inside the weak spot and the growth rate of spherulite increases to the initial level [1].

If the pocket of melt occluded by spherulites is small, the negative pressure may be insufficient for the rupture of the structure and the material solidifies without a break, but with frozen internal stresses. Nowacki et al. [5] reported that the large weak spots tended to cavitate at lower negative pressure than smaller ones, which resulted from an increased probability of the presence of a cavitation nucleus inside the confined melt. It was also found that the shape of the cavities in polypropylene depended on the temperature of crystallization—at low temperatures, the voids were spherical; at high temperatures (i.e., >135 °C), the voids were elongated (similar to cracks), formed along interspherulitic boundaries [5, 6]. Voiding in weak spots was not observed in thin films of polymers crystallized with a free surface because the deficiency of the crystallizing material was compensated here by the thinning of the film. The cavitation during crystallization usually did not occur when the process was non-isothermal because the spherulites were small, a typical size of weak spots was also small, the time of crystallization was short, and the value of negative pressure was below the limit of melt strength.

The above discussed experiments were focused on the studies of cavitation in thin films of crystallizing polymers [5–9]. Galeski and Piorkowska [2] examined volume samples of polypropylene which were crystallized isothermally at 125 °C. They confirmed the existence of weak spots by means of infrared microscopy and the relaxation of stresses after cavitation using Raman spectroscopy. In the second experiment, with the same polymer crystallized in non-isothermal conditions, the authors observed the formation of large, 1- to 2-mm, cavities when the temperature inside the thick specimen decreased much slower than the temperature of the skin layer. Similar conditions often exist in the central parts of the injected PP shapes, where—if the time of solidification is long enough—large cavities visible even by the naked eye are formed. The pressure inside these cavities, detected by a glow discharge from residual gases under high-frequency electromagnetic field, was <0.1 kPa (i.e., <0.1 % of atmospheric pressure) [2].

It is known that non-isothermally crystallized semicrystalline polymers can cavitate during deformation [10, 11]. This happens only in tensile mode, never during compression. Usually, prime voids are generated around the yield point [12, 13]. We showed that there is a competition between two processes possible at yield in the semicrystalline polymer: plastic deformation of crystals by a chain-slip mechanism and the breaking of the amorphous phase (cavitation) [10]. If the polymer crystals are defected, including many dislocations, then with an increase of force, the initiation of plastic deformation of the lamellae is more probable than the break of the amorphous phase. The phenomenon of cavitation in such a polymer is not observed. If lamellae in the polymer are thick, with a reduced number of defects, then the strength of the amorphous phase is lower than the stress of initiation plastic deformation of crystals and cavitation occurs. It was shown that the same kind of polymer, depending on the crystallization and testing conditions (i.e., strain rate, temperature), might cavitate or not [13, 14].

The voids, if present, play an important role during the plastic deformation of semicrystalline polymers. The rapid change of local stress due to void formation stimulates the deformation processes in surroundings crystals, making the deformation of crystals easier. It is visible as a decrease of yield stress, more intensive fragmentation of the lamellae, and an increase of heat generated in the cavitating polymer, when compared with the same kind of material which does not cavitate [10, 15].

Usually, the studies of isothermal crystallization are performed on small pieces of thin polymeric films, and this is a reason why information about the mechanical properties of polymers crystallized in this way is limited. The ductile–brittle transition of isotactic polypropylene and its blend with poly(ethylene-*co*-octene), controlled by isothermal crystallization, was studied by Pang et al. [16]. The mechanical properties of the samples, crystallized at 130 °C, were poor, with breaking at strain of 9 %, immediately after the yield. Cavities were not visible in non-deformed samples.

Some studies of the mechanical properties of isothermally crystallized linear polyethylene were performed by Kennedy et al. [17] who analyzed the influence of molecular weight on tensile properties and brittle–ductile transition. The narrow fractions of polyethylenes with molecular mass below 10^5^ g/mol were brittle when crystallized at 125–130 °C, but showed transitional properties when their molecular mass was larger. Crystallinity was usually high, e.g., 91 % for polyethylene with *M*_w_ = 1.39 × 10^5^ g/mol, crystallized at 125 or 130 °C.

To my knowledge, there was no report on studies on cavitation during the tensile deformation of polymers crystallized isothermally, containing some micrometer-sized cavities due to the crystallization process. It is not even known whether nano-voids accompany micro-voids in an isothermally crystallized polymer. The main subject of this work was to observe nano-cavitation during deformation in isothermally and non-isothermally crystallized polyolefins: high-density polyethylene and polypropylene.

## Experimental

### Materials and methods

The polypropylene used in these studies was Malen P, F401 (*M*_w_ = 297,200 g/mol, *M*_n_ = 56,400 g/mol, MFR = 3 g/10 min (at 190 °C, 2.16 kg)), produced by Basell Orlen Polyolefins. The second polymer, high-density polyethylene (HDPE) Lupolen 6021D (*M*_w_ = 1.8 × 10^5^ g/mol, *M*_w_/*M*_n_ = 7.2), was produced by BASF. The isothermal crystallization process and the crystallinity of the materials were studied using differential scanning calorimetry (DSC). Thermal Analysis TA 2000 apparatus was used to determine the total time of crystallization. Polymers for DSC studies were formed into 0.5-mm-thick films using a hot laboratory press. Pieces weighing 8–9 mg were cut from these films, encapsulated, and crystallized isothermally in the DSC apparatus after melting.

**Fig. 1:**
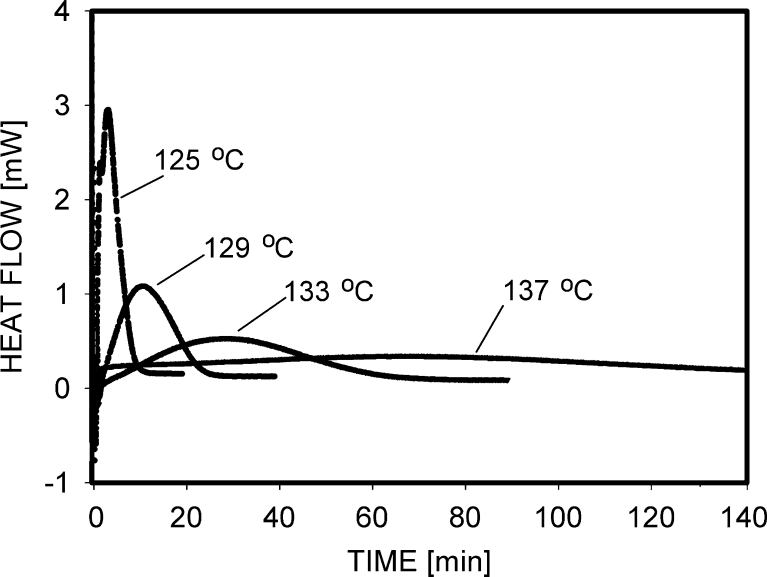
Heat flow as a function of time during isothermal crystallization of PP samples at selected temperatures: 125, 129, 133, and 137 °C

The crystallization of polypropylene was performed at temperatures of 125, 129, 133, and 137 °C. The aim of the measurements was to determine the crystallization time adequate for samples dedicated to studies of mechanical properties. The dependences of heat flow on time for PP samples are shown in Fig. 1. It was observed that in each case, the crystallization process was finished after some time, increasing with the temperature of crystallization. Table 1 shows the times and temperatures which were selected as appropriate for the crystallization of samples designed for future studies.

**Table 1:** Times of crystallization determined from DSC studies as appropriate for the crystallization of samples designed for mechanical studies

Polypropylene		High-density polyethylene	
Crystallization temperature (°C)	Time of crystallization (min)	Crystallization temperature (°C)	Time of crystallization (min)
125	20	122	15
129	30	125	30
133	60	129	>90
137	100		

The DSC apparatus was also used to determine the crystallization times for high-density polyethylene. The studies were done at crystallization temperatures of 122, 125, and 129 °C. The crystallization times selected for the preparation of HDPE samples designed for mechanical tests are shown in Table 1. The crystallization process was very slow for a sample remaining at 129 °C, so I concluded that this temperature was too high for sample preparation because there was a risk of thermal degradation.

The DSC technique was used to determine the melting temperature, crystallinity, and the mean lamellae thickness of polypropylene. The measurements were done when the samples were heated at the rate of 10 K/min. The heat of melting equal to 165 J/g [7] for polypropylene was assumed in the calculations of crystallinity degree. The mean thickness of the lamellae, *L*_m_, was determined using Eq. 1.

1 $$ {L_{\text{m}}} = 2 \times {\sigma_e} \times {T_{\text{mo}}}/\left( {{H_{\text{f}}} \times {\rho_{{\text c} }} \times \left( {{T_{\text{mo}}} - {T_{\text{m}}}} \right)} \right) $$

where *σ*_e_ is the surface energy, *T*_mo_ the equilibrium melting temperature, *H*_f_ the heat of fusion, *ρ*_c_ the density of the crystalline phase, and *T*_m_ is the temperature of melting. The following values were taken for calculations: *σ*_e_ = 62.3 × 10^−7^ J/cm^2^, *T*_mo_ = 208 °C [7], *H*_f_ = 165 J/g [7], and *ρ*_c_ = 0.936 g/cm^3^. Similar calculations were done for high-density polyethylene specimens, assuming the heat of fusion per unit volume as $\Delta {H_{\text{f}}} = {H_{\text{f}}} \times {\rho_c} = { }280{\text{ J}}/{\text{c}}{{\text{m}}^3}$ [18], *σ*_e_ = 4.4 × 10^−6^ J/cm^2^ [19], and *T*_mo_ = 415 K [19].

Samples for a mechanical test were prepared using a hot stage constructed in my laboratory. The main elements of the hot stage were two copper blocks containing heaters. The heaters and thermometers (resistors) were placed inside the blocks whose temperature was controlled by two programmable 535 Process Controllers (Omega, USA). A thin (0.5 mm) film of polymer was placed between two microscopic glasses; such a sandwich was positioned between the heating blocks. The hot stage with the sample was fixed in a polarizing microscope, replacing an original microscopic stage. Two small holes drilled inside the blocks gave a possibility to observe the growth of the crystalline structures inside the examined film. The polypropylene film was heated at the rate of 10 °C/min to the temperature of 220 °C, kept 3 min at that temperature, and cooled down to the final crystallization temperature, again at the rate of 10 °C/min. The crystallization temperature for polypropylene was 125, 129, 133, or 137 °C. The growth of spherulites was observed during solidification, and it was concluded that the images were invariable after times of 20, 30, 60, and 100 min; therefore, after these times, the samples were cooled to room temperature. The aforementioned times of crystallization were the same as determined by DSC (Fig. 1).

Some PP samples were also crystallized non-isothermally, with the aim of comparing the properties of differently prepared materials. In this case, the polypropylene film was melted at 220 °C, and after 3 min of conditioning, the temperature of the stage was linearly decreased to 25 °C, with the cooling rate of 5 °C/min. This rate ensured that the crystallization process was not very fast and that the polypropylenes having relatively large and not much defected lamellae should be able to cavitate during deformation. Table 2 presents a list of the isothermally crystallized PP samples prepared for mechanical studies.

**Table 2:** List of samples studied in mechanical and SAXS tests

Sample name	Polymer	Crystallization conditions
PP-N	Polypropylene	Non-isothermal, melting at 220 °C, 3 min, and cooling 5 °C/min
PP125		Isothermal, 125 °C, 20 min
PP129		Isothermal, 129 °C, 30 min
PP133		Isothermal, 133 °C, 60 min
PP137		Isothermal, 137 °C, 100 min
PE-N	High-density polyethylene	Non-isothermal, melting at 190 °C, 2 min, and cooling 4 °C/min
PE122		Isothermal, 122 °C, 25 min
PE125		Isothermal, 125 °C, 40 min

High-density polyethylene films were prepared similarly to PP films. The 0.5-mm-thick foils, formed by the hot press, were melted inside the heating stage, conditioned for 2 min at *T* = 190 °C, and cooled down at the rate of 10 °C/min to one of two crystallization temperatures: 122 or 125 °C. At these temperatures, the film was kept for 25 or 40 min, respectively. The structures growing in polyethylene were small, the evolution of morphology poorly visible with the microscope, and the end of crystallization difficult to determine. This was the reason why the crystallization times were a bit longer than recommended by the DSC observations (see Table 1), remembering that the DSC measurements were done in similar, but not exactly the same, conditions. Moreover, the longer time should result in the thickening of crystals, which would increase the possibility of a cavitation.

Part of the polyethylene samples was also crystallized in non-isothermal conditions. In this case, the polymer film was melted inside the crystallizing device at 190 °C; after 2 min of conditioning at this temperature, the samples were cooled down at the rate of 4 °C/min to the final temperature of 100 °C. Microscopic observations confirmed that the crystallization was finished before reaching the final temperature, so the film was next cooled down to room temperature and removed from the apparatus.

It is known that the process of isothermal crystallization examined in the microscale depends on temperature. For PP and HDPE, three crystallization regimes are distinguished, and the architecture of the solidified polymers depends on these regimes [7, 20, 21]. All the polypropylene samples studied here were crystallized in regime III and the HDPE samples crystallized in regime II.

Samples for the tensile test were cut from the aforementioned films. They had a dog bone shape with an initial gauge zone length of 12.5 mm, width of 4.1 mm, and thickness of 0.3–0.5 mm (PP) or 0.5–0.6 mm (HDPE). Table 2 describes the symbols used in the text and the crystallization procedures for polymer samples examined by the tensile test and by X-ray scattering measurements.

The mechanical properties of polymers were studied at room temperature using an Instron 5582 tensile machine. Five samples of each kind were tested in uniaxial drawing. The rate of deformation was 5 %/min (i.e., 8.3 × 10^−4^ s^−1^) for HDPE and 2 %/min (i.e., 3.3 × 10^−4^ s^−1^) for PP. Black marks were drawn on the surface of each specimen. Changes of the initial 1-mm distances between the marks were recorded using a Canon D50 digital photo camera. At the same time, the actual sample width was recorded. My samples were too thin for accurate measurements of thickness by the photo method, so it was assumed that the thickness changed proportionally to the changes of width.

The local strain of a sample, *ε*, was determined using Eq. 2.

2 $$ \varepsilon = { }\left[ {\left( {L - {L_{{0}}}} \right)/{L_{{0}}}} \right] \times 100{ }\left[ \% \right] $$

where *L*_0_ = 1 mm is the initial distance between the black marks and *L* is the distance between the marks in the deformed sample. Similarly, volume strain, Δ*V*, may be defined as

3 $$ \Delta V = { }\left( {V - {V_{{0}}}} \right)/{V_{{0}}} $$

where *V*_0_ is the initial volume and *V* is the actual volume. Both these volumes were calculated from the width of the sample, its thickness (assumed proportional to the width), and the distance between the black marks, all measured for the most deformed part of the sample. The proportionality of width and thickness changes was controlled for selected strains.

Some mechanical experiments were performed using a laboratory tensile machine, constructed in the Hasylab Laboratory in Hamburg. The technical characteristic of this device was similar to Instron, but with the applied force limited to 100 N. This machine was movable, so it was possible to place the stretched sample on the way of the X-ray beam from the DORIS III synchrotron source (A2 beamline) and conduct X-ray scattering measurements in situ during deformation. Radiation, with a wavelength of 0.15 nm, scattered under small angles (SAXS) was detected by a MarCCD 2D detector. SAXS patterns were later used to detect cavities and to determine crystalline structure parameters. It was possible to detect nano-cavities with dimensions below 80 nm. The times of a single pattern registration were 24 s for PP and 6 s for HDPE. The CCTV camera connected to a monitor and computer helped in the observation and recording of distances between the marks on the specimen surface and in the observation of the X-ray beam position on this surface. Before deformation, the beam was positioned in place where the beginning of plastic deformation could be expected. If the neck was formed in the expected illuminated area, then the results of the test were analyzed. The examined PP samples were deformed to break, which happened quickly after the yield point. Mechanical tests of HDPE specimens were stopped at the engineering strains of 90–110 % because in this polymer the most interesting phenomena occur earlier, around the yield point.

On the SAXS pattern representing the scattering from the non-deformed semicrystalline polymer, only one maximum of intensity, resulting from the crystalline periodic structure, is usually visible. This maximum was used to determine a long period of structure based on the dependence of *Is* ^2^ = *f*(*s*), where *I* is the intensity and *s* is the scattering vector [22]. For comparison, the long periods were also determined by a correlation function method [23, 24]. With this method, the contributions from the amorphous and crystalline phases were separated and their thickness calculated. The evidence of the cavitation process, when examined by SAXS, is a large increase of the scattering intensity. When the concentration of the cavities is small and they are dispersed in the polymer matrix, the radius of gyration, *R*, can be determined from the equation

4 $$ I(s){ } = {I_0}{\rho^2}{v^2}\exp ( - 4{\pi^2}{s^2}{R^2}/3) $$

where *I* is the intensity of scattering, *I*_0_ is the initial intensity of the beam, *ρ* the difference of electron density between the void and the matrix, *v* the volume of scattering objects, *s* the scattering vector, and *R* is the gyration radius [25]. If the scattering objects are randomly oriented, then the radius does not depend on the direction of measurement and may be used for the determination of the size of the cavities if their shape is known. Otherwise, *R* depends on the direction and only rough information about void sizes is provided.

The wide-angle X-ray scattering (WAXS) camera was used for observations of possible crystallographic transformations. A source of CuK_α_ radiation, operating at 30 kV and 50 mA, was used. Two-dimensional scattering images were recorded by a camera equipped with a Kodak imaging plate. The distance between a sample and a recording plate was 5 cm. Exposed imaging plates were analyzed with a PhosphorImager SI system (Molecular Dynamics). The stretching of the samples was interrupted at a selected strain and the sample fixed in a special frame, which preserved the state of the strain. The frame with the sample was then placed in a holder of WAXS apparatus. Typical time of acquisition was 7 min. For some samples, the WAXS patterns were recorded after releasing the mechanical stress with the aim of detecting eventual changes of diffraction patterns.

The spherulitic morphology of polypropylene was studied using the polarized light microscope. Thin (20 μm) slices were cut from polymer films with a microtome equipped with a glass knife. This procedure was not applied for polyethylene because the expected size of spherulites (based on crystallization observation) was too small for exact microscopy observations.

## Results and discussion

### Polypropylene

The crystalline structures of polypropylene films, crystallized according to the procedures described in Table 2, were determined using the DSC and SAXS methods and observed by the polarized light microscopy. The long period of lamellar structure was calculated both from the maximum on the SAXS intensity curve and from the correlation function. The latter method also gives information about the thickness of crystalline and the amorphous components. Results of the SAXS experiments are collected in Table 3.

**Table 3:** Results of SAXS and DSC experiments for non-deformed PP samples

Temperature of crystallization (°C)	SAXS			DSC			
	Long period from intensity curve (nm)	Long period determined from the correlation function (nm)	Lamellae thickness determined from the correlation function (nm)	Temperature of melting, max (°C)	Lamellae thickness (nm)	Heat of melting (J/g)	Crystallinity (%)
Non-isothermal	15.6	14.6	9.3	165.3	9.1	108	65
125	17.6	16.7	11.3	164.4	8.9	115	69
129	19.1	18.5	11.8	165.3	9.1	117	71
133	19.7	18.1	12.4	166.9	9.4	120	73
137	20.7	18.6	12.0	168.2	9.8	121	73

Table 3 shows that the long period of polypropylene structure depends on the crystallization temperature. It was the smallest (15.6 nm) for non-isothermally crystallized PP and increased with isothermal crystallization temperature to 20.7 nm at *T* = 137 °C. A similar tendency was observed when the long period was calculated from the correlation function. In all the examined materials, the crystalline layers were thicker than the amorphous ones and lamellae thickness increased with the crystallization temperature.

The second source of information on the crystalline structure of polypropylene was the DSC technique. The results of these studies are also presented in Table 3, where the following parameters are shown: temperature of melting (maximum of peak), lamellae thickness, heat of melting, and crystallinity.

The DSC thermogram of the non-isothermally crystallized PP sample shows that the melting peak was wide, with a maximum at 165.3 °C. Melting peaks in the isothermally crystallized specimens were narrower. The shift of position of the maximum in the direction of the higher temperature was observed with the increase of crystallization temperature. Calculations of the lamellae thickness (Eq. 1) showed its increase with the crystallization temperature. It follows from Table 3 that the heat of melting is higher at higher temperatures, which means an increase of crystallinity from 69 to 73 %, when the temperature of isothermal crystallization changes from 125 to 137 °C. Non-isothermally crystallized PP samples had a lower crystallinity of 65 %.

The main conclusion from the data in Table 3 is that crystals are thicker and crystallinity is higher when the temperature of crystallization increases. The observed differences in lamellar thickness are the result of the specifics of the applied DSC and SAXS methods. The different methods applied for thickness measurements were compared some years ago by Zhou and Wilkes [26]. Previously, I studied the properties of the same polymer, but prepared with a different cooling procedure during crystallization [15]. The comparison of the results showed that if the PP film was slowly cooled from melt, then its melting temperature was 163.1 °C and the heat of melting is 90.9 J/g. If the same material was quickly cooled in water, the melting temperature decreased to 162.2 °C and the heat of melting was only 82.0 J/g. In both cases, the process of solidification was faster than for the samples discussed in this manuscript.

**Fig. 2:**
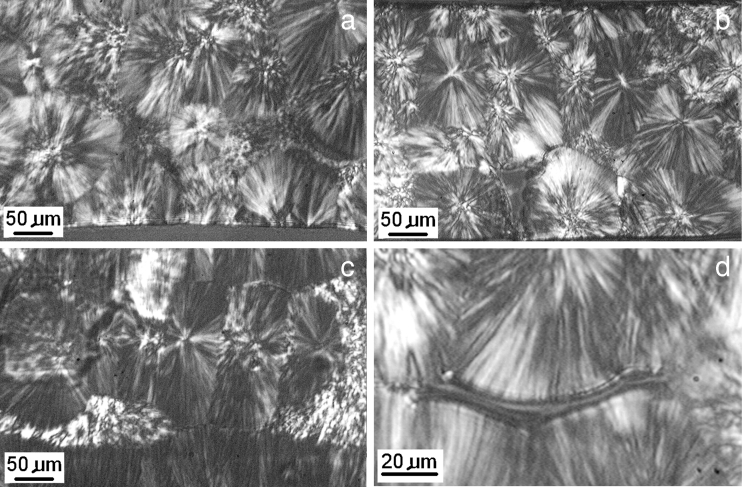
Morphologies of the polypropylene samples crystallized: non-isothermally (**a**), isothermally at 125 °C (**b**), isothermally at 133 °C (**c**), and a fragment of the structure of sample PP125, with a long void visible on the spherulitic border (**d**)

Figure 2 shows exemplary images of 20-μm-thick polypropylene slices observed with the polarized light microscope. These slices represent cross-sections of the non-deformed PP films. The supermolecular structure, with large spherulites, is clearly visible on all photographs. Some of the spherulites were nucleated on the film surfaces (at the bottom of Fig. 2a or c), where the local temperature during cooling from *T* = 220 °C to the crystallization temperature was lower than in the volume of the specimen. This promoted the nucleation of spherulites on the surface. The nucleation was a little bit more frequent in the samples crystallized at higher temperatures (Fig. 2c), however not so intensive to dominate the structure formation. The spherulites were bigger in the samples crystallized isothermally, but the increase of size with temperature was moderate. In the samples crystallized isothermally, where the time of crystallization was longer, the formation of weak spots was observed, with breaking of the structure in the largest of them. The exemplary weak spot is visible in the lower part of Fig. 2b. The elongated void, formed along the spherulites borders, is shown in Fig. 2d.

**Fig. 3:**
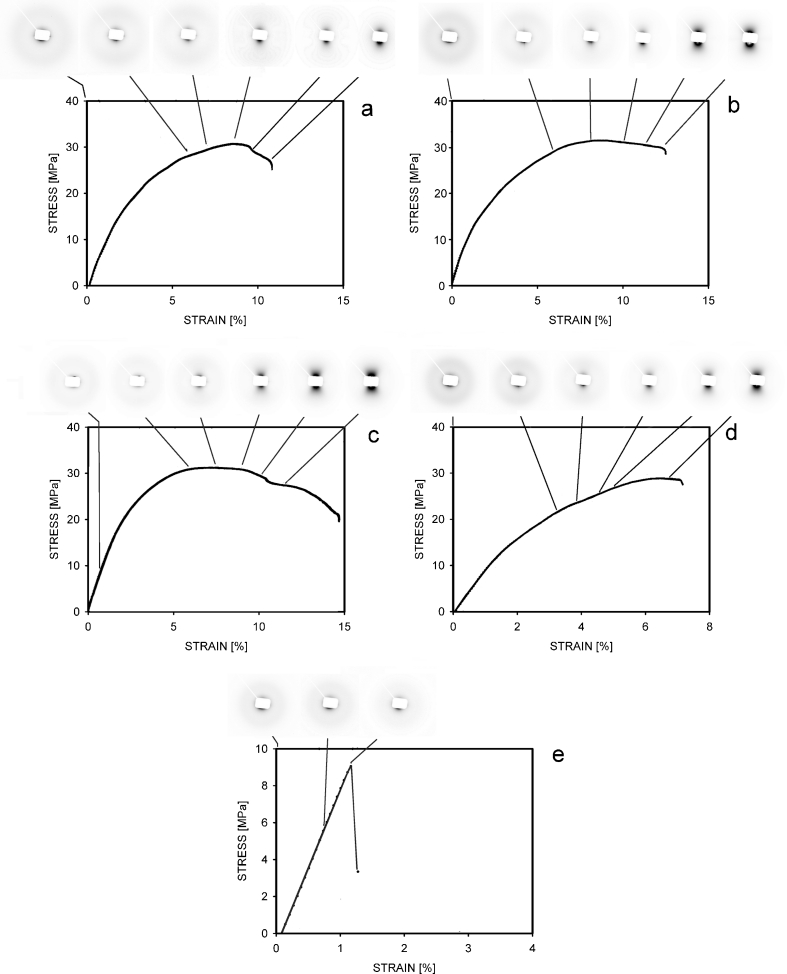
SAXS patterns and stress–strain curves for polypropylene samples crystallized in the following conditions: non-isothermal (**a**), 125 °C (**b**), 129 °C (**c**), 133 °C (**d**), and 137 °C (**e**). The strains for which the patterns were registered are indicated by *lines*. The deformation direction on patterns was vertical

The results of the tensile test of PP samples are shown in Table 4; representative stress–strain curves are presented in Fig. 3. The samples crystallized non-isothermally and at lower crystallization temperatures of 125 and 129 °C were able to deform plastically with yield; however, the structure broke shortly after the yield. The specimens crystallized at higher temperatures broke before the yield point. Elongation to break decreased with an increase of crystallization temperature, and for the samples crystallized at 137 °C, it was only 1.5 %. The samples crystallized at temperatures of 133 and 137 °C broke before the yield point. The elastic modulus, calculated from the stress at 1 % of strain, increased slowly with the crystallization temperature. The mechanical properties of the examined samples were not satisfactory for many applications, but the deterioration of some mechanical properties in more crystalline material is a well-known phenomenon, observed for isothermally crystallized PP also by Pang et al. [16].

**Table 4:** Mechanical properties of PP samples deformed in tension at the rate of 2 %/min

Crystallization conditions (°C)	Elastic modulus (GPa)	Yield stress (MPa)	Yield strain (%)	Stress at break (MPa)	Strain at break (%)
Non-isothermal	1.3	31	9	29	13
125	1.2	32	8	30	12
129	1.3	32	7	30	8
133	1.3	-	-	27	6
137	1.4	-	-	9	2

Simultaneously with the results of the mechanical test, the small-angle X-ray scattering patterns were recorded. The goal of these observations was to detect cavities. Figure 3 shows the stress–strain curves measured for individual samples and the accompanying X-ray patterns recorded in situ during tensile drawing. The samples with maximum available strains were examined here with the aim of getting maximum information on scattering and cavitation, so the strains to break on curves in Fig. 3 differ from the average values given in Table 4.

In the patterns representing the non-deformed polypropylene, only the scattering from periodic crystalline structure is visible. It is the evidence of an important fact that nano-voids are not formed in PP during isothermal crystallization. The very small black dots visible near the beam stop are traces of the non-completely covered initial beam. In some images representing the deformed samples, dark spots representing the scattering on cavities are visible. The first voids arise when the deformation is close to the yield. It happens for the samples crystallized non-isothermally and for the samples crystallized isothermally at 125 and 129 °C. Polypropylenes which were crystallized at higher temperatures cavitated earlier, before the yield point (see Fig. 3d, e). It follows from Fig. 3e that first voids in the PP 137 specimen were formed at a strain of 1.3 % only. The vertical orientation of the dark spots in scattering images is the evidence that the nano-sized voids were elongated horizontally, i.e., perpendicularly to the deformation direction which was vertical.

**Fig. 4:**
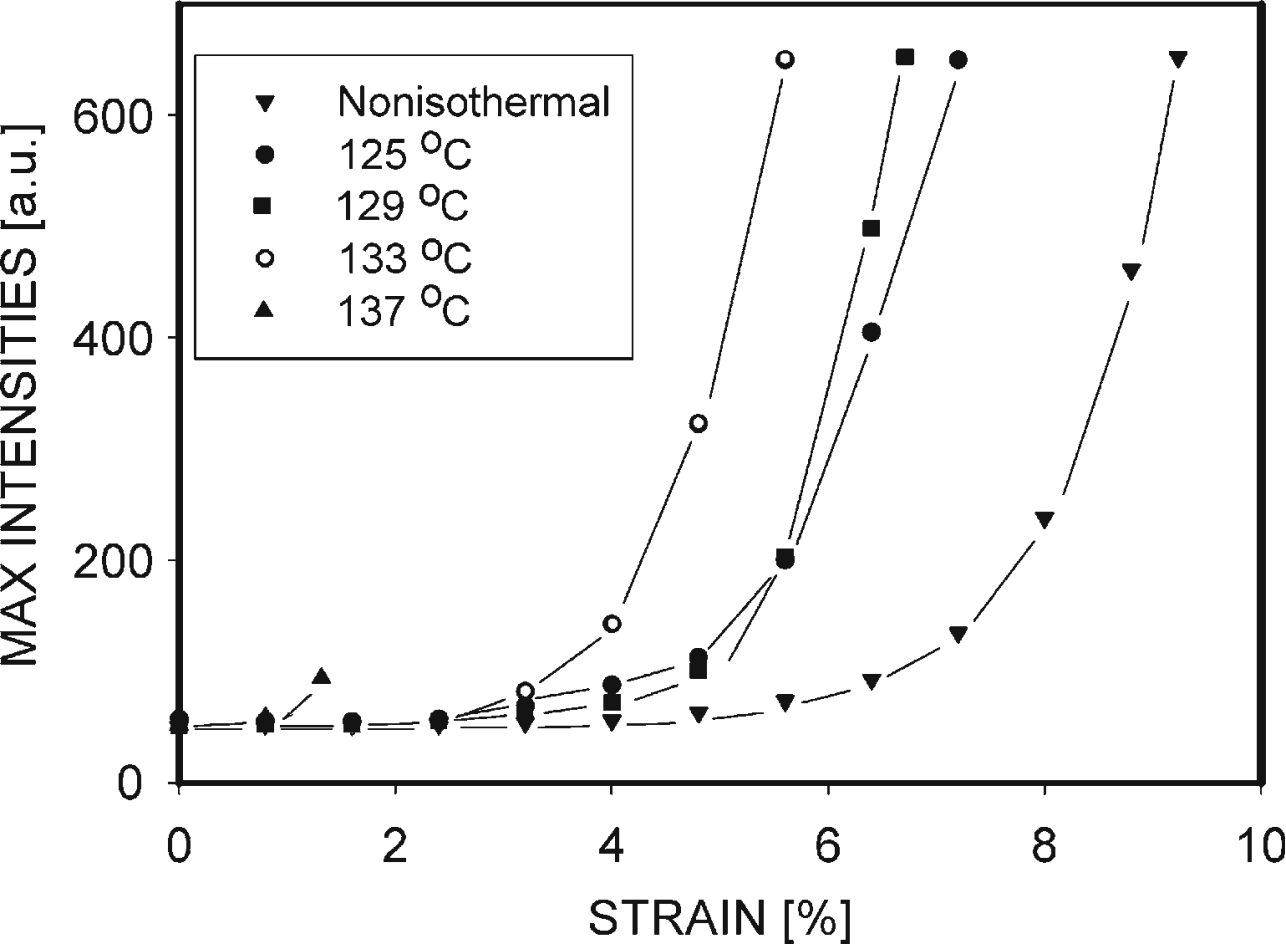
Maximal intensities in SAXS patterns of the deformed PP samples

From the patterns in Fig. 3, it is difficult to determine exactly the beginning of cavitation. For this goal, it may be useful to analyze maximal intensities in scattering images as a function of deformation. These intensities should quickly increase from the moment when the first nano-cavities are formed. Figure 4 presents relations between the strain and the maximal intensities visible on patterns in Fig. 3. A significant increase of intensity (more than 10 %) was observed for the non-isothermal PP-N sample when the strain reached 6.5 %. In the samples crystallized isothermally, such an increase was noticed earlier: at 4 % for PP 125 and PP 129 specimens, 3 % for PP 133, and only 1.3 % for PP 137. In each case, the rise of intensity happened before the yield point. It means that cavitation preceded a large-scale plastic deformation of crystals occurring at yield. Especially the polypropylenes crystallized at temperatures of 133 and 137 °C cavitated very early. The curve representing PP 129 is not precisely in the middle between the curves for PP 133 and PP 125. A possible explanation is that for the PP 129 sample, the X-ray beam was not located exactly in the position where the cavitation was initiated first. The voids in beam position were formed here a moment later, with a time shift, which is seen in Fig. 4 as an increase of intensity at a larger strain.

**Fig. 5:**
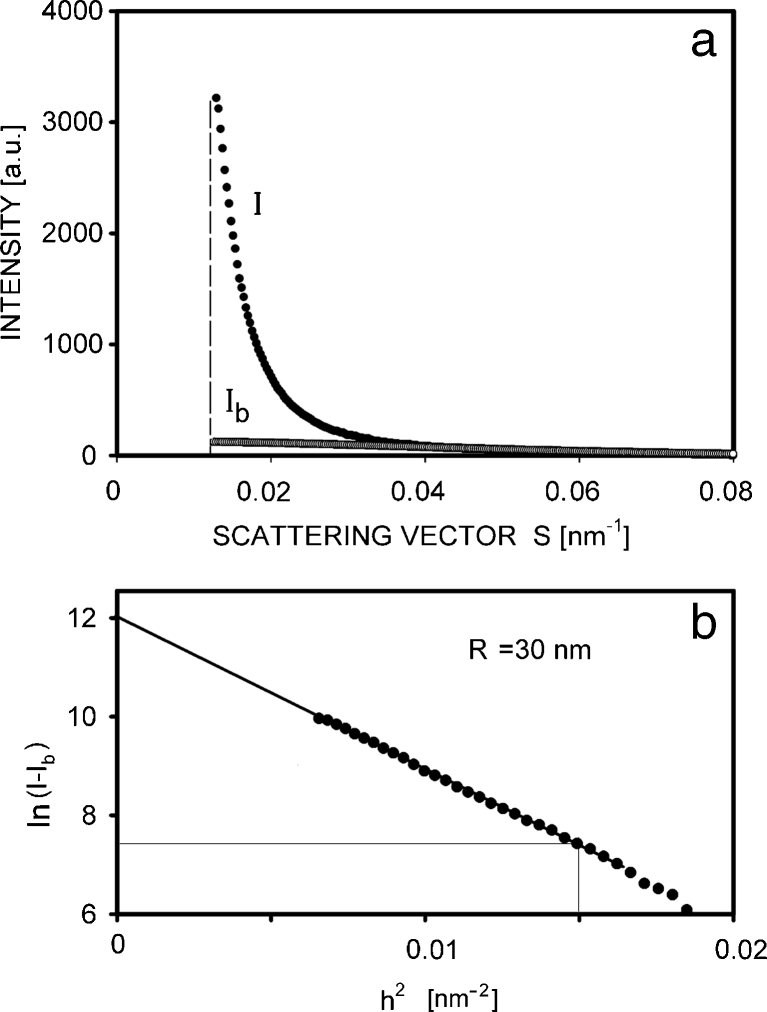
**a** Profiles of intensities measured in vertical direction in SAXS patterns shown in Fig 4, representing the non-isothermal PP sample before deformation, *I*_b_, and after deformation to yield strain, *I*. **b** Logarithmic dependence of the scattering intensity, *I* − *I*_b_, as a function of the square of the *h* vector (*h* = 2*πs*). This dependence was used for the determination of the gyration radius, *R*

There is no general method for the determination of void sizes directly from the SAXS results; however, this size may be characterized by calculating the gyration radius, *R*, done according to Eq. 4. I analyzed the vertical profiles of the intensities from the scattering patterns, registered at the yield point. The background of scattering, resulting from the crystalline periodic structure, was subtracted, assuming its shape to be similar to that of the non-deformed sample. The procedure of the determination of the gyration radius from Eq. 4 is illustrated in Fig. 5 on the example of non-isothermally crystallized PP sample. The following values of *R* were found: 30 nm for the non-isothermal sample, 28 nm for PP 125, 42 nm for PP 129, and 34 nm for PP 133. It means that systematic changes of the gyration radius with crystallization conditions were not found. Sample PP 137 was broken much before the yield; however, some limited cavitation was observed inside it, characterized by a 50-nm radius of gyration.

**Fig. 6:**
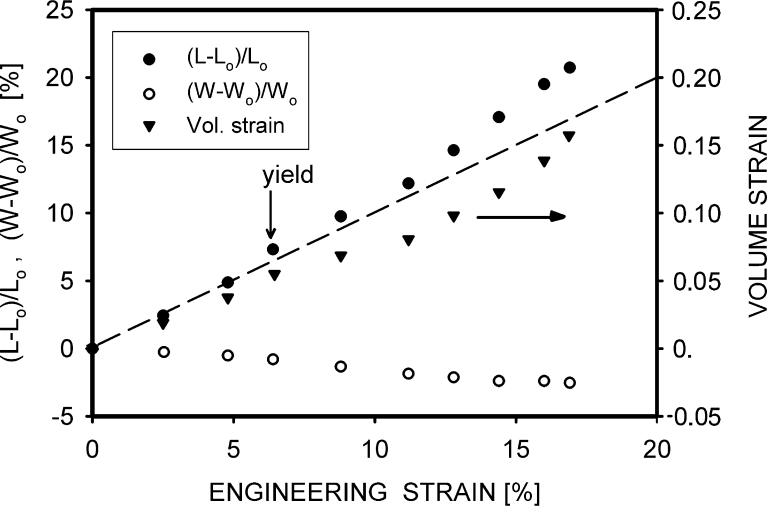
Local strain, (*L* – *L*_0_)/*L*_0_, and width, (*W* – *W*_0_)/*W*_0_ (in percent) vs. engineering strain, measured in the most deformed part of the PP129 sample. *L* is the actual and *L*_0_ the initial length between marks; *W* and *W*_0_ are the actual and initial widths in the neck area, respectively. The *dashed oblique line* shows where the local strain is equal to the engineering strain

The uniformity of deformation and shape of the polypropylene samples are retained to the yield point. Beyond it, the deformation concentrates in a small volume where a neck is formed. Because of this localization, changes of the local dimensions are largest in the neck, where the local strain exceeds the macroscopic strains. These facts are illustrated in Fig. 6, where the dimensions of the sample, represented by the local strain, (*L* – *L*_0_)/*L*_0_, and width, (*W* – *W*_0_)/*W*_0_, are shown as a function of engineering strain. They were determined in the position where the neck was formed. The distance between the marks drawn on the surface of the sample (i.e., 1 mm) was a basis for local strain determination. This particular sample, presented in Fig. 6, was crystallized at 129 °C, and its deformation to break was larger than typical for this kind of material (i.e., strain of 8 %; see Table 4). A continuous declination of the local strain data from a straight line since the beginning of yield (i.e., 6 % of strain) is clearly visible. The increase of length is accompanied by a decrease of the sample width. If the decrease is not proportional to the length increase, the local volume of the sample changes. For the PP 129 specimen, the increase of volume was observed for all strains.

The increase of volume before the yield is usually attributed to elastic deformation. In many cases, this increase is small and the accurate measurements of volume are difficult. A much larger increase of volume is often observed during the plastic deformation phase. Cavities formed inside the amorphous phase are responsible for it. Fortunately, for the PP129 sample, it was possible to measure correctly its dimensions up to the moment of break at 17 % of strain. I found that the 2D (i.e., defined as length × width^2^) volume strain at this deformation was equal to 0.16.

It was more than the value of 0.1 determined previously for specimens of the same material, which were formed from the air-cooled melt [13]. The other PP specimens, crystallized in different conditions, broke around the yield, and the experimental error of measurements was too large to determine the volume correctly.

### Polyethylene

The second examined polymer was high-density polyethylene. The initial properties of HDPE are presented in Table 5 which shows the data of the long period and lamellae thickness, all determined in SAXS measurements. The long periods for the isothermally crystallized specimens were larger than for those crystallized in non-isothermal conditions. Also, the lamellae were thicker in the isothermally crystallized samples (24.4 nm) when compared with the non-isothermal PE-N (19.9 nm).

**Table 5:** Properties of non-deformed HDPE samples determined by the SAXS and DSC methods

Temperature of crystallization (°C)	SAXS			DSC			
	Long period (*Is* ^2^ vs. *s*) (nm)	Long period from the correlation function (nm)	Lamellae thickness from the correlation function (nm)	Temperature of maximum of melting peak (°C)	Lamellae thickness from Eq. 1 (nm)	Heat of melting (J/g)	Crystallinity^a^ (%)
Non-isothermal	31.4	27.8	19.9	135.9	21.9	236.9	82
122	34.6	30.8	23.8	136.7	25.3	240.0	83
125	33.3	32.1	24.4	136.9	26.3	243.7	84

^a^Assuming the heat of fusion as 289 J/g [36]

The crystalline properties of HDPE have also been studied using the DSC technique; the results of the measurements are shown in Table 5. This table shows a small increase of the melting temperature, heat of melting, and crystallinity when comparing PE-N with PE122 and PE125. Bassett showed that polyethylene lamellae are planar when the crystallization temperature is higher than 127 °C [27]. A lower crystallization temperature produces spherulites whose lamellae have C- or S-shaped profiles, in which the angle between the lamellar normal and *c*-axis changes continuously, but with a maximum at ~35° [27, 28]. The values of lamellae thickness measured by SAXS were slightly different from those calculated from the DSC data, which resulted from the specifics of both methods; however, the general tendency—increase of thickness with the temperature of crystallization—was preserved.

From the data in Table 5 and from previous observations [29, 30], I expected that cavitation would appear in the samples crystallized isothermally and probably in the non-isothermal ones too. When the same polyethylene was solidified in air, it had 21.1-nm-thick lamellae and cavitated during stretching. However, if the molten HDPE samples were cooled in cold water, then thinner lamellae grew (13.9 nm) and the polymer did not cavitate [29].

Similarly to polypropylene, the evolution of morphology during crystallization was observed in polyethylene by a polarized light microscope. The spherulitic structures were formed in all HDPE samples crystallized from melt. Spherulites were fine, with diameters of a few micrometers only, so details of the structure were difficult to observe. The cavitation during crystallization was not visible.

**Fig. 7:**
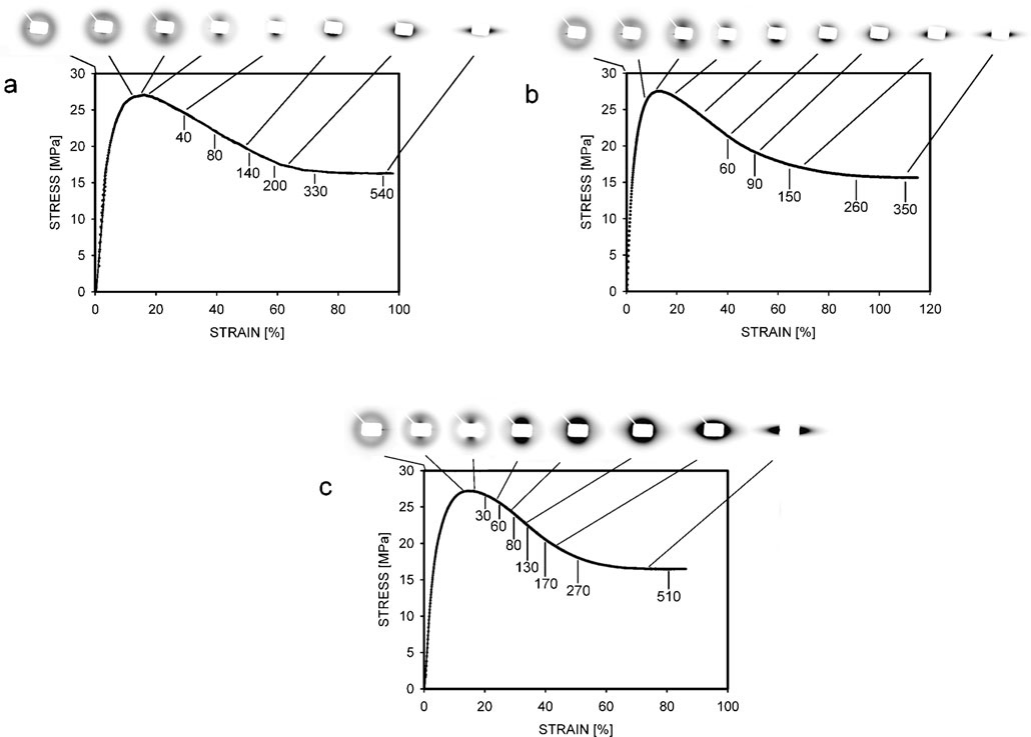
Stress–strain curves and SAXS patterns registered at selected strains for HDPE samples: non-isothermally crystallized (**a**), crystallized at the temperature of 122 °C (**b**), and crystallized at the temperature of 125 °C (**c**). The *numbers* show local strains in percent. The deformation direction on the patterns is vertical

The mechanical properties of the polyethylene samples were tested during tensile drawing; the results are shown in Table 6 and Fig. 7. I found that the elastic modulus of the isothermally crystallized specimens was 12–14 % larger than the modulus of PE-N. All samples were able to deform beyond the yield point. The values of yield stress were similar for the examined specimens, in the range of 27.0–27.4 MPa. Analysis of the yield strains showed that yield was reached earlier in the samples with larger crystals (i.e., PE125 and PE122). Elongations to break were not determined because mechanical tests were stopped after reaching plateau on the stress–strain curve, i.e., at the engineering strain of 90–120 %, which was much before the break point of the examined materials (>400 %).

**Table 6:** Mechanical properties of polyethylenes

Crystallization conditions (°C)	Elastic modulus (GPa)	Yield stress (MPa)	Yield strain (%)
Non-isothermal	0.84	27.0	13.3
122	0.96	27.4	12.5
125	0.94	27.3	11.6

Parts of the HDPE specimens were tensile tested with a simultaneous registration of scattered X-ray radiation. Examples of the stress–strain curves and related scattering patterns are shown in Fig. 7. The deformation of HDPE was non-uniform and the neck was formed in all samples. Local strains in the neck were larger than macroscopic engineering strains. This is illustrated in Fig. 7, where the numbers near the curves show the values of local strain. Like polypropylene, the SAXS patterns for the non-deformed samples contain only the contribution from scattering on the crystalline periodic structure. It means that cavities with sizes of 2–80 nm were absent in the isothermally crystallized HDPE specimens before deformation. The intensive scattering on cavities is clearly visible in the deformed samples. It is first observed shortly before the yield, at strains of 11–12 %.

The patterns recorded beyond the yield are oriented in the deformation direction (i.e., vertically in Fig. 7), which means that the cavities were elongated perpendicularly to the deformation direction. At some local strain (in the range of 100–200 %), the shape of the pattern changes. This is interpreted as a reorientation of voids into the deformation direction. From the evolution of the patterns, it may be deduced that voids were more elongated at higher strains. The changes of void shapes were similar to those observed previously for HDPE [29, 30], when the samples were slowly solidified in air or were annealed.

**Fig. 8:**
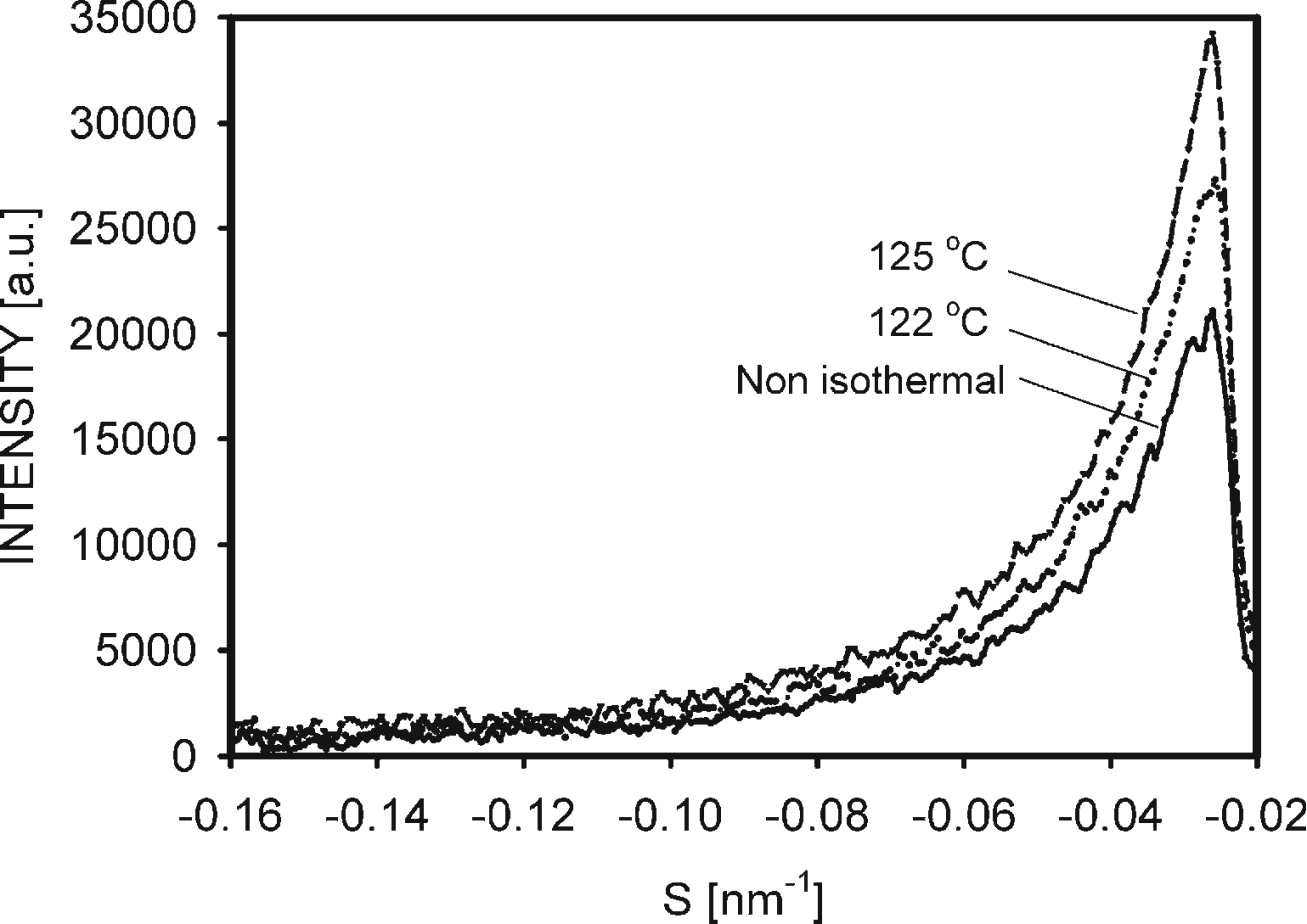
Scattering profiles taken in the vertical direction from patterns in Fig. 7, representing samples deformed to an engineering strain of 25 %. The decreasing intensity on the *right side* indicates the edge of the beam stop. *S* is the scattering vector

The X-ray scattering on voids was most intensive for the PE125 sample; however, the differences in intensities between specimens are difficult to estimate from Fig. 7. It will be helpful to analyze the scattering profiles. Figure 8 presents the intensity profiles taken in the deformation direction for samples deformed to 25 % of the engineering strain, i.e., shortly after the beginning of cavitation. It is clear that the most intensive scattering was from the PE125 sample and the lowest intensities were measured for the PE-N specimen.

In addition to the profiles in Fig. 8, total scatterings from the samples, registered by a Mar CCD detector, were determined. Darkening of the scattering pattern was analyzed using the ImageJ 1.41 software (public domain, W. Rasband, National Institute of Mental Health, USA). It was done for specimens deformed to engineering strains of 25 %, i.e., when the cavitation was relatively intensive, but the local strains in the samples were comparable. The performed analysis showed that if scattering from the non-isothermally crystallized sample was normalized to 1.0, then it was 1.18 for PE122 and 1.42 for PE125. These results confirm that at the beginning of plastic deformation, the scale of cavitation strongly depends on the previous crystallization process.

An important aspect of the cavitation process is the size of cavities because, together with the number of voids, it affects the behavior and properties of the material. It is known that the intensity of scattering is proportional to the square of the total volume of scattering objects. Guinier’s approach (Eq. 4) may be used to calculate the size if the voids are dispersed in the polymer [25], which happens at the beginning of cavitation. Computations of the radius of gyration were carried out for samples deformed to 20 % of the engineering strain. Here, the vertical profiles of intensities were analyzed, taken from the scattering patterns in Fig. 7. Because the scattering from voids dominated, it was assumed that the background and scattering from the crystalline structures were on the same level as the scattering from the non-deformed polymer and both components were subtracted from the total scattering. The calculations showed that only one population of nano-voids existed in the HDPE samples. The radius of gyration was equal to 14.8 nm for the non-isothermally crystallized HDPE, 16.7 nm for the same polymer crystallized at 122 °C, and 16.5 nm for the HDPE sample crystallized at 125 °C.

**Fig. 9:**
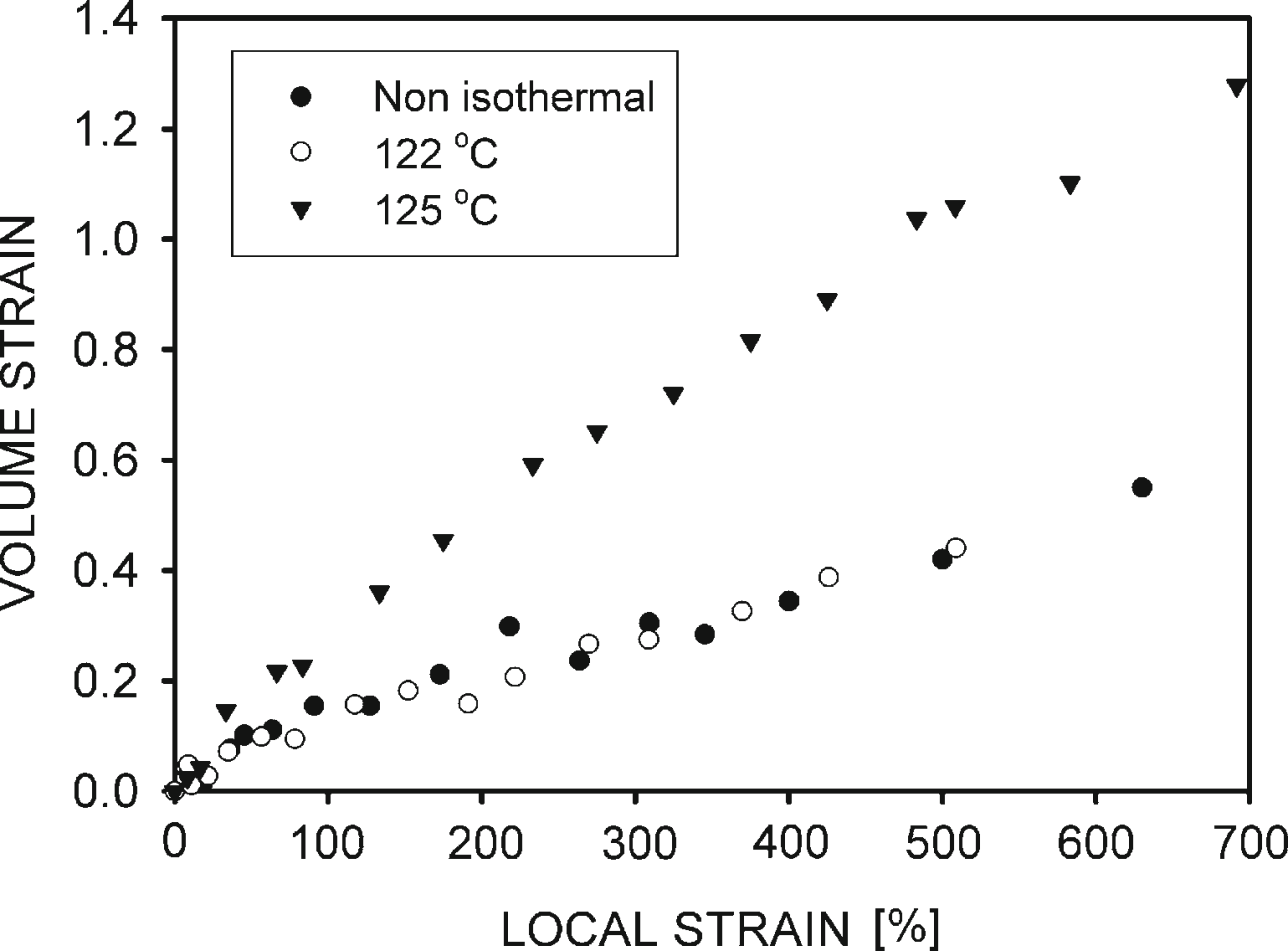
Dependence of volume strain ((*V* – *V*_0_)/*V*_0_) on the local strain for polyethylene samples

Cavitation is the process in which a new volume is created. Thus, the measurement of volume increase is the way to characterize the scale of cavitation. Changes of the volume strain (defined by Eq. 3) as a function of the local strain are shown in Fig. 9. The presented dependences were registered for individual samples and represented a typical behavior for particular crystallization temperatures. However, it should be mentioned that data for the individual samples within the set may be a little different from typical because the volume increase is sensitive to the degree of localization of deformations in the neck and the neck shape was not exactly the same for the compared samples. The samples in which the neck was well developed immediately after the yield had a larger volume strain than the samples where neck formation was a longer process.

A significant increase of volume was observed in all samples, beginning from the yield point. The volume strain was similar for the PE-N and PE122 specimens, but a much stronger increase was observed for PE125, where the volume of the analyzed element was even twice as big as the original one.

**Fig. 10:**
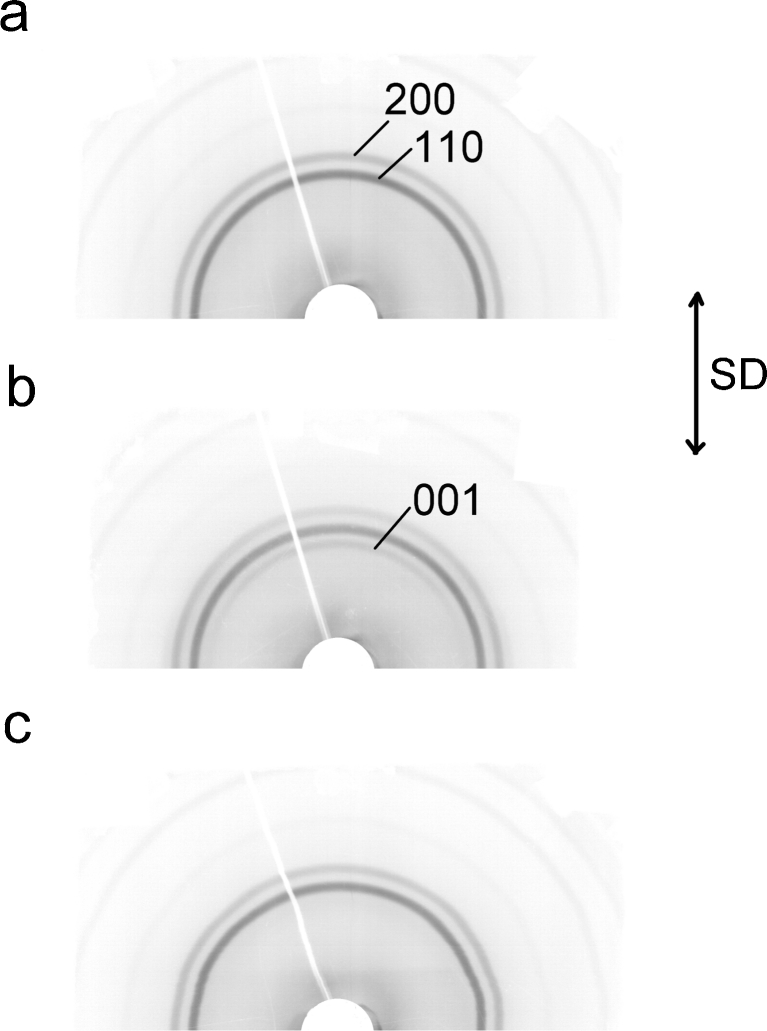
WAXS patterns registered for the PE122 sample before deformation (**a**), after deformation to yield point (**b**), and after deformation to yield and release of stresses with free ends (**c**). The main diffracting crystallographic planes are indicated. *SD* is the direction of deformation

It is known that not only nano-voids but also micrometer-sized voids are responsible for the volume increase. Voids of this size scatter light, and when the material is observed in reflected light, it is whiter than the initial one. The presence of micrometer-sized voids in the examined PE was confirmed by whitening which occurred at yield. I excluded from the consideration the second potential reason of whitening, i.e., additional crystallization of the strained polymer, because the DSC studies did not show an increase of crystallinity for the deformed polymer. WAXS observations showed changes in crystalline polymorphs with deformation. Two-dimensional patterns were registered for the non-deformed samples (Fig. 10a), for the same sample deformed to yield (Fig. 10b), and after stress release (Fig. 10c). Two circles, which showed up in the WAXS patterns for the undeformed material and for strained to yield, are associated with a diffraction from (110) (inner circle) and (200) (outer circle) lattice planes of orthorombic form. In the samples under preserved strain at yield, a third ring—inside the (110) ring—appears visible, indexed as (001) of monoclinic form. This form is a result of the martensitic transformation of the orthorhombic form under stress. The monoclinic form is not visible in the pattern at such strain if the sample was relaxed with free ends. The reversibility of transition is not accompanied by a similar effect of whitening, which excludes crystallographic changes from the factors responsible for whitening.

**Fig. 11:**
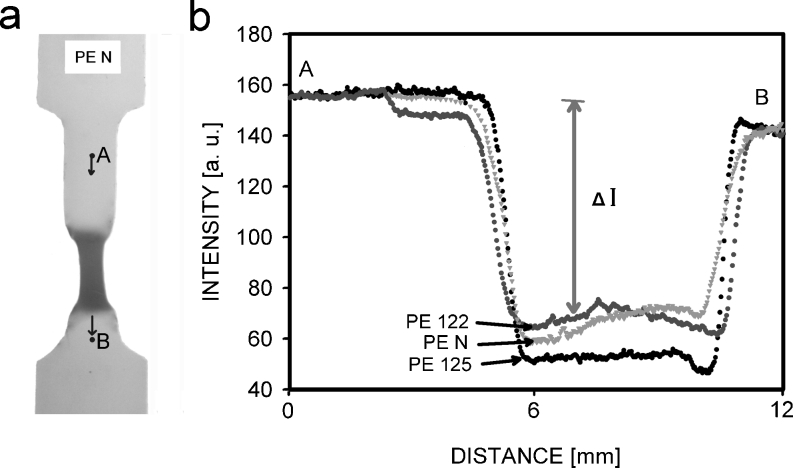
PE-N sample illuminated by light going through the sample (**a**) and intensity of light transmitted through the sample, measured along the line between points *A* and *B* for PE-N, PE122, and PE125 (**b**)

An example of the whitening effect for the deformed sample is presented in Fig. 11a. It is the PE-N specimen observed in transmitted light after deformation. The source of light was the Image Eraser (produced by Molecular Dynamics), usually used for erasing imaging plates. This equipment gives uniform white light on a large surface. The objects of investigations were polyethylene samples deformed to plateau on the stress–strain curve, with similar length of the neck and with similar thickness after deformation, in the range of 0.29 ± 0.01 mm measured in the central part of neck. The samples were placed on the transparent plate and photographed. Intensive scattering of the transmitted light in the neck part of the specimen is visible in the photographs as a dark area. In reflection mode, the same area is seen as being whiter. The observations showed that the scale of light scattering depended on the sample. Figure 11b shows the profiles of intensities taken from point A to point B for the PE-N sample (see Fig. 11a) and by the same way for the rest of my specimens. The most intensive scattering in the neck was for the PE125 sample. Scattering for two other samples was on a similar level. If the mean values of the gray levels on the photographs determined for the neck area using the ImageJ software are compared, the relations of intensity decreases, Δ*I*, are 1.0:1.02:1.12 for PE122, PE-N, and PE125, respectively. This is in good agreement with measurements of the volume in Fig. 9, where similarities for PE-N and PE122 were visible, but the volume for PE125 was much bigger. The results of volume strain and light and X-ray scatterings show that nano- and micro-voids are most intensive in PE125. In the PE122 sample, the nano-voids are more frequent than in PE-N; however, the volume increase is similar for PE122 and PE-N, which means that micro-voids are more frequent in PE-N or the frequencies are similar, but the contribution from micro-voids dominates in the volume strain.

## Conclusions

The experiments performed with two polymers—polypropylene and high-density polyethylene—showed that plastic deformation of these materials occurs with cavitation. Their mechanical properties and the process of cavitation depend on the conditions at which the polymer was crystallized.

Although both polymers were crystallized isothermally, their morphologies were different. In polyethylene, small (a few micrometers in diameter) spherulites grew, so also small weak spots were formed between them and micrometer-sized cavities were not formed during crystallization [5]. The SAXS studies showed that nano-voids were not present in the non-deformed HDPE. The cavitation happened when the external force was applied to the HDPE specimen. Voids were observed for the first time before the yield point, at strains of 1–2 % below the yield strain.

The DSC and SAXS measurements confirmed that crystallinity and the crystal thickness of polyethylene increased with the temperature and time available for crystallization. It is known that higher crystallinity and thicker, less defected crystals favor the cavitation during deformation [10]. These resulted in the most intensive cavitation (both nano- and micrometer-sized) observed in polyethylene samples crystallized at the highest temperature. The nano-voids were at yields elongated perpendicularly to the deformation direction, which indicates that they were formed between the lamellae inside spherulites, not on the spherulitic borders or not inside the weak spots. Inside the spots and on the borders, more random orientation and less elongated voids are expected. Moreover, some observations of the isothermally crystallized samples showed that melting inside weak spots occurred at lower temperatures than melting of surrounding spherulites, which means that the matter inside these spots is less organized, with more defected crystals. The conditions for cavitation inside weak spots are worse than those existing inside the spherulites.

The elongated shapes of the SAXS patterns mean that the radius of gyration, *R*, is not directly related to the real dimension of voids and depends on the measurement direction. In current studies, the radius of gyration in the vertical direction was 15–17 nm.

Evolution of the shape of nano-cavities with elongation was similar in all samples and resembled those studied previously [30]. The voids were initially elongated perpendicularly to the deformation direction, but this orientation changed for large strains, where cavities were elongated in the deformation direction. The change was forced by the reorganization of the surrounding lamellae, just as it was described in previous papers [13, 14, 29, 30]. The small voids were accompanied by larger, micrometer-sized voids. Their presence was confirmed by the whitening of material which occurred after yield.

The internal structure of the second examined polymer, polypropylene, was different because larger spherulites were grown during crystallization. Morphological observations showed that large weak spots, with diameters of 10–100 μm, were formed between spherulites in the isothermally crystallized films. In some of them, the polymer cavitated near the end of crystallization, forming micrometer-sized voids. The SEM photographs showed that weak spots were not very frequent. Nowacki et al. [5] observed that the negative pressure buildup was slower in large weak spots formed in polypropylene specimens crystallized at the highest temperatures. The reason was some relaxation of stretched molecules possible during long crystallization and the reduced viscosity of melt. This resulted in less frequent micro-cavitation observed during crystallization in the highest temperatures, although the weak spots were larger. The SAXS studies showed that the large voids in PP were not accompanied by smaller, nanometer-sized voids.

The crystals in PP samples were thick, so it was supposed that tensile deformation would occur with cavitation, which was confirmed experimentally for all the examined samples. When the polymer was deformed, voids were observed for the first time before the yield point. In polypropylene, the cavitation began earlier (i.e., at smaller strains) for these samples which were crystallized at higher temperatures. The DSC and SAXS measurements confirmed that the crystallinity and crystal thickness of polypropylene increased with the temperature and time available for crystallization. It is known that more regular chain folding in higher crystallization temperatures results in a decrease in the number of tie molecules [31]. These molecules connecting adjacent crystallites play an important role in the mechanical performance of polymers [32, 33]. A limited number of the molecules bridging lamellae cannot preserve the continuity of the amorphous phase when the polymer is stretched. This is a possible reason of the early cavitation observed in these polypropylene specimens which were formed at the highest temperatures.

The SAXS patterns confirmed that nano-cavities in PP were elongated perpendicularly to the drawing direction. The intensities of scattering at yield, depending on the scale of cavitation, were larger for the specimens crystallized at higher temperatures. The volume strain for the PP129 sample was larger than those reported previously for the same polymer crystallized when cooling was in air [12–14].

Polypropylene spherulites usually are built from the lamellae of α-crystallographic form, creating a so-called cross-hatching structure. Previous studies of the cavitation in α- and β-allotropic modifications of PP showed that the scale of voiding was larger in the β-form, where this structure is not present, but only the radial lamellae growth [14]. Norton and Keller [34] and Janimak et al. [35] found that the cross-hatching density in the isothermally crystallized PP is reduced with temperature and that radial lamellae dominate at higher crystallization temperatures. Therefore, the arrangement of lamellae crystallized at a higher temperature resembles that characteristic for β-form specimens, and it should result in more intensive cavitation during deformation. This is a reason, aside from the decreasing number of tie molecules, why the amorphous phase is weaker and a more intensive cavitation is observed in the samples crystallized at higher temperatures.

The size of the cavities was estimated from the radius of gyration. The samples which were crystallized in controlled isothermal conditions had values of *R* (i.e., 28–50 nm) larger than those found previously for PP crystallized by another cooling procedure (13–18 nm). The larger values of *R* were not associated with the increase of the amorphous layer thickness.

The above observations led to the conclusion that cavitation is more intensive when the crystallinity is higher and crystals are thicker, which happens during isothermal crystallization at high temperatures. Comparison of the cavitation during deformation in polypropylene containing some micro-voids resulting from crystallization and in polyethylene without such voids shows that the nano-voiding process occurs similarly, i.e., without visible influence of preexisting micro-voids. Those micro-voids are present in the interspherulitic areas, separated from the interlamellar amorphous layers inside spherulites, where the new nano-voids are generated during deformation. The large weak spots and big voids disturbing the internal distribution of stresses are probably responsible for the poor tensile properties and the early break of PP samples.
